# Innate-immune crosstalk orchestrates T cell-mediated rejection in kidney transplants

**DOI:** 10.3389/fimmu.2026.1827224

**Published:** 2026-07-07

**Authors:** Yuyun Hu, Zhiqiang Chen, Yujun Liang, Zhixuan Wu, Yijian Zhang, Junyu Guo, Chunqiang Dong, Guanmiao Chen, Michael Williams, Emily Johnson, Min He, Wei Du, Boqian Wang

**Affiliations:** 1 Department of Nephrology, The First People’s Hospital of Nanning, Nanning, China; 2 Department of Nephrology, Yuebei People’s Hospital Affiliated to Shantou University Medical College, Shaoguan, Guangdong, China; 3 The Second Hospital, Cheeloo College of Medicine, Shandong University, Jinan, Shandong, China; 4 Wuming Hospital Affiliated to Guangxi Medical University, Nanning, China; 5 Organ Transplant Center, General Hospital of Northern Theater Command, Shenyang, China; 6 Pathology Department, General Hospital of Northern Theater Command, Shenyang, China; 7 Department of Organ Transplantation, The First Affiliated Hospital of Guangxi Medical University, Nanning, China; 8 Department of Basic Science, YuanDong Life California Ivy Research Institute, West Hollywood, CA, United States; 9 Department of Urology, Zhujiang Hospital of Southern Medical University, Guangzhou, China

**Keywords:** biomarkers, CD8+ T cells, immune microenvironment, kidney transplantation, single-cell RNA sequencing, T cell–mediated rejection

## Abstract

**Objective:**

T cell–mediated rejection (TCMR) remains a major barrier to long-term kidney allograft survival, driven by recipient immune responses against donor antigens. A comprehensive understanding of the cellular and molecular mechanisms underlying TCMR is essential for developing novel diagnostic and therapeutic strategies.

**Methods:**

We integrated two public single-cell RNA sequencing datasets (GSE145927 and E-MTAB-12051) to construct a comprehensive single-cell atlas of kidney allograft biopsies. Unsupervised clustering, functional scoring, trajectory inference, gene regulatory network analysis, and cell–cell communication analysis were performed. Key findings were validated in a murine TCMR model.

**Results:**

In the single TCMR sample analyzed, we observed several cell populations that were enriched and may be associated with TCMR. An NQO1^+^NDUFS4^+^ proximal tubular subset exhibited marked activation of oxidative phosphorylation and fatty acid metabolism, consistent with metabolic remodeling under inflammatory stress. An S100A8^+^ macrophage subset displayed a pro-inflammatory phenotype and actively recruited CD8^+^ T and NKT cells via chemokine signaling. A CCL4L2^+^ NKT subset exacerbated rejection by enhancing immune recruitment and cytotoxic functions. These innate immune cells formed extensive communication networks with adaptive immune cells through chemokine (CXCL10-CXCR3) and inflammatory (TNF-TNFRSF1A/B) axes, suggesting a potential molecular basis for innate-adaptive immune crosstalk. Additionally, a DUSP1^+^ effector CD8^+^ T subset was selectively enriched in TCMR and showed robust T cell receptor activation, and may be associated with graft injury potentially through the B2M/MHC class I axis. In a murine model, we observed upregulation and functional dependence of Dusp1^+^ CD8^+^ T cells, consistent with the findings from the single human TCMR case.

**Conclusion:**

This hypothesis-generating study, based on a single TCMR case, provides a single-cell atlas of the TCMR immune microenvironment and suggests several subsets and pathways that may be involved. Our findings suggest that innate immune cells may initiate and amplify adaptive responses through chemokine and inflammatory networks, providing new insights into the potential role of innate immunity in allograft injury. The DUSP1^+^ effector CD8^+^ T cell–B2M/MHC class I axis was observed in this single case and may represent a candidate for further investigation.

## 1 Introduction

Kidney transplantation is widely recognized as one of the most effective renal replacement therapies for patients with end-stage renal disease, significantly improving long-term survival and quality of life (1, 2). However, long-term graft survival remains severely limited by immune-mediated rejection, which continues to be a major barrier to further improvements in clinical outcomes after kidney transplantation (1). Following allogeneic transplantation, the recipient immune system recognizes donor-derived antigens and initiates a complex cascade of immune responses, ultimately resulting in graft injury and even graft loss (3). From a clinicopathological perspective, T cell–mediated rejection (TCMR) and antibody-mediated rejection (ABMR) represent the two major forms of immune injury after transplantation, among which TCMR is the most common manifestation of acute rejection (2).

Clinical studies have demonstrated that TCMR is not only highly prevalent but also markedly heterogeneous. Approximately 40% of patients with TCMR respond poorly to standard anti-rejection therapies, and recipients diagnosed with TCMR by biopsy exhibit significantly reduced long-term graft survival (4). Indeed, most irreversible graft dysfunction ultimately occurs in patients with refractory or recurrent TCMR, underscoring its central pathogenic role in the progression of allograft injury. The development of TCMR depends on the processing and presentation of donor antigens by antigen-presenting cells, followed by recognition by recipient T cells through the T cell receptor (TCR). This initiating event triggers T cell activation, clonal expansion, and effector differentiation, driving the recruitment of multiple immune effector cells into the graft, thereby establishing a sustained inflammatory microenvironment that damages tubular and interstitial structures (5).

In this process, CD8+ T cells are recognized as key effector cells potentially mediating graft injury. Activated CD8^+^ T cells shape the inflammatory environment by secreting pro-inflammatory cytokines including IFN-γ and TNF-α, thereby potentially contributing to immune rejection. Increasing evidence suggests that CD8^+^ T cells are not a functionally homogeneous population; instead, their differentiation states, activation levels, and molecular characteristics are closely associated with the occurrence, severity, and clinical outcomes of TCMR (6). However, the functional specialization, dynamic evolution, and intercellular interactions of distinct CD8^+^ T cell subsets within the TCMR-specific pathological microenvironment remain insufficiently characterized.

Single-cell RNA sequencing (scRNA-seq) provides a powerful tool to address these challenges. This technology enables high-resolution characterization of immune cell composition and transcriptional states within complex tissues, revealing cellular heterogeneity and functional states that are difficult to detect using bulk transcriptomic approaches. Through systematic single-cell–level dissection of the transplant-associated immune microenvironment, scRNA-seq has emerged as an essential approach for elucidating the mechanisms underlying transplant rejection (7).

Based on this background, the present study aimed to systematically analyze immune cell composition and functional states under TCMR conditions using kidney transplant–related single-cell transcriptomic datasets, with a particular focus on key immune cell subsets and signaling pathways closely associated with TCMR pathology. By integrating single-cell molecular features with clinicopathological phenotypes, we sought to explore the immunological features of TCMR and to generate hypotheses for future identification of diagnostic biomarkers and therapeutic targets.

## 2 Materials and methods

### 2.1 Data collection and processing

Two publicly available human scRNA-seq datasets were included in this study: GSE145927 (8) and E-MTAB-12051 (9), which were obtained from the Gene Expression Omnibus (https://www.ncbi.nlm.nih.gov/geo/) (10) and the ArrayExpress database (https://www.ebi.ac.uk/biostudies/arrayexpress) (11), respectively.

Dataset inclusion criteria were predefined as follows: studies involving human kidney transplant recipients; samples derived from kidney allograft biopsy tissues; outcomes related to immune rejection after kidney transplantation; and the use of scRNA-seq technology to generate high-resolution gene expression data. Accordingly, datasets focusing on non-renal organs or non-transplant-related diseases, animal model studies unrelated to human kidney transplant outcomes, and datasets lacking appropriate control groups or unsuitable for reliable comparative analysis were excluded.

Regarding dataset composition, GSE145927 contains whole-exome sequencing data from peripheral blood mononuclear cells (PBMCs) and scRNA-seq data from kidney allograft core biopsy tissues, encompassing donor-specific antibody (DSA)–positive ABMR and acute kidney injury (AKI) without definitive rejection (DSA-positive or DSA-negative). E-MTAB-12051 includes scRNA-seq data from kidney allograft biopsy samples representing DSA-positive ABMR, AKI without rejection (DSA-positive or DSA-negative), and T cell–mediated rejection (TCMR).

After integrating the two datasets, the final cohort comprised six DSA-negative non-rejection samples, eight DSA-positive non-rejection samples, six DSA-positive ABMR samples, and one TCMR sample.

Quality control was performed to filter low-quality cells, potential doublets, and dying cells. Specifically, the number of detected genes (nFeatures), total transcript counts (nCounts), and the proportion of mitochondrial gene expression were assessed for each sample. Cells with gene or transcript counts within the top or bottom 1% of the distribution, as well as cells with mitochondrial gene expression exceeding 10%, were excluded. High-quality cells retained after filtering were used for downstream analyses.

### 2.2 Data normalization and atlas construction

Processed scRNA-seq data were analyzed using the Seurat 4.1 (12), including normalization, unsupervised clustering, and differential gene expression analysis. SCTransform was applied to normalize gene expression matrices and stabilize variance, minimizing the effects of sequencing depth and technical noise. Batch effects between datasets were corrected using the Harmony 1.0 algorithm. Shared nearest neighbor (SNN) graphs were constructed based on principal component analysis, and cells were clustered using the FindClusters function to identify transcriptionally similar cell populations.

Uniform manifold approximation and projection (UMAP) (13) was applied for dimensionality reduction and visualization of clustering results to illustrate transcriptional heterogeneity among cell populations.

Cluster-specific marker genes were identified using the FindAllMarkers function, comparing each cluster with all others. Differentially expressed genes with p < 0.05 were considered statistically significant. Cell clusters were annotated into known major cell types based on established canonical markers from previous single-cell studies.

On this basis, secondary clustering was performed for specific cell populations to resolve internal heterogeneity and identify distinct subpopulations. Marker genes for each subcluster were identified and used to assign biological identities.

### 2.3 AUCell gene set enrichment analysis

To quantify the functional states of renal epithelial and immune cell populations, AUCell analysis (14) was performed at the single-cell level. Enrichment scores were visualized using boxplots.

### 2.4 Functional enrichment analysis

To systematically investigate the biological functions of identified cell subsets in TCMR, functional enrichment analyses were performed using the clusterProfiler 4.1 (15). Gene Ontology (GO) biological process and Kyoto Encyclopedia of Genes and Genomes (KEGG) pathway enrichment analyses were conducted based on marker genes for each subset. Gene set enrichment analysis (GSEA) was further performed using MSigDB gene sets (16) (c5.bp.v7.4.symbols.gmt and c2.cp.kegg.v7.4.symbols.gmt). A pvalue < 0.05 was considered statistically significant.

### 2.5 Pseudotime trajectory analysis

Monocle 3 was used to perform pseudotime trajectory analysis to reconstruct potential cell state transitions. Dimensionality reduction was conducted using reduceDimension, followed by cell ordering with orderCells. UMAP visualization was applied to depict cell distributions along pseudotime trajectories.

### 2.6 Gene regulatory network analysis

Gene regulatory networks were inferred using the SCENIC framework (14). Co-expression modules were identified, direct targets were inferred using cisTarget, and regulon activity was quantified. Results were visualized using the ComplexHeatmap package (17).

### 2.7 Cell–cell communication analysis

Cell–cell interactions were analyzed using the CellChat 1.5. Based on normalized gene expression and cell type annotations, ligand–receptor interactions were inferred using the CellChatDB database (including checkpoint, cytokine, growth factor, and other signaling families). Communication networks were quantified and visualized using CellChat’s built-in functions (18).

### 2.8 Animal model construction

Male C57BL/6J (H-2b) and BALB/c (H-2d) mice (8–10 weeks, 22–26 g) were housed under SPF conditions (10 C57BL/6J and 20 BALB/c mice). All procedures were approved by the Animal Medical Research Ethics Subcommittee of the General Hospital of the Northern Theater Command (Approval No. 2026-51). A fully MHC-mismatched kidney transplantation model was established with three groups: syngeneic control (BALB/c donor → BALB/c recipient, n=5 per side), allogeneic transplantation (C57BL/6 donor → BALB/c recipient, n=5 per side), and allogeneic transplantation with MHC-I blockade (C57BL/6 donor → BALB/c recipient, n=5 per side) receiving intraperitoneal injections of anti-MHC-I neutralizing antibody (clone 28-14-8, Bio X Cell, cat. No. BE0077, USA) at a dose of 250 μg per mouse on day -1 (one day before transplantation) and on postoperative days 1, 3, and 5.

Donor kidneys were harvested after cold heparinized saline perfusion; the renal artery and vein were anastomosed end-to-side to the recipient abdominal aorta and inferior vena cava with 10-0 sutures, and the ureter was implanted into the bladder, leaving both native kidneys intact and without immunosuppression. Mice were euthanized on postoperative day 5 for graft collection; humane endpoints (severe wound dehiscence, major hemorrhage, infection/abscess, respiratory distress, cyanosis, shock, or moribundity) triggered immediate exclusion. Euthanasia was performed by intraperitoneal injection of an overdose of pentobarbital (150 mg/kg), and death was confirmed by cessation of respiration and heartbeat.

### 2.9 Quantitative real-time PCR

Total RNA was extracted from mouse kidney samples using SparKZol Reagent (SparKJade, China) according to the manufacturer’s instructions. cDNA was synthesized using HyperScript™ III RT SuperMix with gDNA Remover (EnzyArtisan, China). Quantitative PCR was performed on a Kubo q225–0388 system using TB Green^®^ Premix Ex Taq™ II (TaKaRa, Japan). The following primer pairs were used: Gapdh (forward: 5′-ctacatggtctacatgttcc-3′, reverse: 5′-ctcctggaagatggtgatg-3′); Dusp1 (forward: 5′-ctccaaggaggatatgaagc-3′, reverse: 5′-gattctgcactgtcaggcac-3′); B2m (forward: 5′-gaccggcctgtatgctatc-3′, reverse: 5′-ggaactgtgttacgtagcag-3′); Gzmb (forward: 5′-catgaagtcaagccccactc-3′, reverse: 5′-cttcacagtgagcagcagtc-3′); Prf1 (forward: 5′-cactcggtcagaatgcaagc-3′, reverse: 5′-gaacctctgtgtgttcactg-3′);Ifng (forward: 5′-ctgtttctggctgttactgc-3′, reverse: 5′-gccagttcctccagatatcc-3′). All reactions were performed in triplicate. Gene expression was normalized to Gapdh and calculated using the 2^^−ΔΔCt^ method.

### 2.10 Western blotting

Total protein was extracted from mouse kidney samples using RIPA lysis buffer supplemented with protease and phosphatase inhibitors. Protein concentration was determined using a BCA protein assay kit. Equal amounts of protein (20 μg per lane) were separated by SDS-PAGE and transferred onto PVDF membranes. The membranes were blocked with 5% non-fat milk in TBST for 1 h at room temperature and then incubated overnight at 4 °C with primary antibodies against GAPDH, PRF1, B2M, GZMB, IFNG, and DUSP1. After washing, the membranes were incubated with HRP-conjugated goat anti-rabbit IgG secondary antibody (1:20,000) for 2 h at room temperature. Protein bands were visualized using an enhanced chemiluminescence detection kit and imaged with a chemiluminescence imaging system. Band intensities were quantified using ImageJ software and normalized to GAPDH. Detailed information on all primary antibodies, secondary antibody, key reagents, and kits used in this study is provided in **Supplementary Tables 1**, **2**.

### 2.11 Statistical analysis

Statistical analyses of animal experiments were performed using GraphPad Prism software. Two-group comparisons were assessed using independent samples t-test, and multiple-group comparisons were performed using one-way analysis of variance (ANOVA) followed by LSD *post hoc* test. Correlation analysis was performed using Spearman’s rank correlation coefficient. Differentially expressed genes were identified using the Wilcoxon rank-sum test, with P-values adjusted by the Benjamini-Hochberg method. All bioinformatics analyses were conducted on the Bioinforcloud platform (http://www.bioinforcloud.com). Data are presented as mean ± standard deviation.

## 3 Results

### 3.1 A global single-cell atlas of T cell–mediated rejection after kidney transplantation

**Figure 1:**
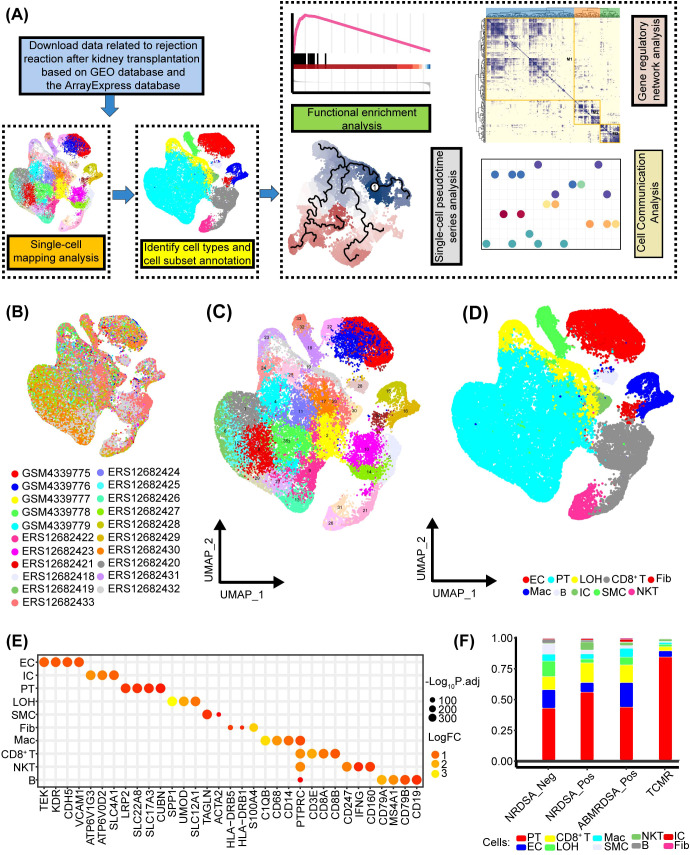
Integrated single-cell transcriptomic landscape of T cell–mediated rejection (TCMR) following kidney transplantation. **(A)** Overview of the study design and computational analysis pipeline. Rejection-related single-cell RNA sequencing (scRNA-seq) datasets were collected from the GEO and ArrayExpress repositories. After preprocessing and quality control, downstream analysis included cell type identification and annotation, functional enrichment, pseudotime trajectory reconstruction, gene regulatory network inference, and cell–cell communication analysis. **(B)** UMAP projection of all cells from individual samples. Each color corresponds to one sample, illustrating data integration and cellular distribution across patients. **(C)** Clustering of integrated single cells reveals 36 transcriptionally distinct clusters. Clusters were annotated based on canonical marker expression. **(D)** Annotated UMAP plot displaying major cell types identified across the rejection landscape, including endothelial cells (EC), macrophages (Mac), loop of Henle epithelial cells (LOH), smooth muscle cells (SMC), proximal tubular cells (PT), B cells, intercalated cells (IC), CD8^+^ T cells, fibroblasts (Fib), and natural killer T (NKT) cells. **(E)** Bubble plot of selected marker genes highlights cell type–specific transcriptional signatures. The size of each bubble represents –log_10_ adjusted P-value for differential expression, and color intensity represents average log fold change (logFC). **(F)** Relative abundance of major cell types in different clinical conditions, including NRDSA-negative, NRDSA-positive, ABMRDSA-positive, and TCMR groups.

The overall study design is illustrated in (**Figure 1A**). Dimensionality reduction and unsupervised clustering were performed on single-cell RNA sequencing data from GSE145927 and E-MTAB-12051, yielding a total of 132,103 high-quality single-cell transcriptomes. Within the constructed single-cell atlas, 36 distinct cell clusters were clearly identified and visualized (**Figures 1B, C**). These clusters were further annotated into 10 major cell types (**Figure 1D**): endothelial cells characterized by specific expression of TEK and KDR; intercalated cells with high expression of ATP6V1G3, ATP6V0D2, and SLC4A1; proximal tubular cells expressing LRP2 and SLC22A8; loop of Henle cells marked by SPP1, UMOD, and SLC12A1; smooth muscle cells expressing TAGLN and ACTA2; fibroblasts characterized by S100A4 and HLA-DRB5 expression; macrophages expressing C1QB, CD68, and CD14; CD8^+^ T cells with high expression of CD3E, CD8A, and CD8B; natural killer T (NKT) cells expressing IFNG and CD160; and B cells marked by CD79A, MS4A1, and CD79B (**Figure 1E**).

Compared with the donor-specific antibody (DSA)–negative non-rejection group, proximal tubular cells were relatively enriched in the TCMR group, whereas the relative abundance of other cell types was reduced. In contrast, compared with the DSA-positive non-rejection group, macrophages, loop of Henle cells, and endothelial cells were relatively increased in antibody-mediated rejection (ABMR), while NKT cells and proximal tubular cells were relatively decreased (**Figure 1F**).

These findings provide a single-cell overview of kidney transplant rejection. However, because only one TCMR sample was available, the observed differences in cellular composition involving the TCMR condition should be considered hypothesis-generating and await validation in independent cohorts.

### 3.2 The NQO1+NDUFS4+ proximal tubular cell subset exhibits activation of oxidative phosphorylation–centered metabolic pathways

During the pathological process of T cell–mediated rejection, proximal tubular epithelial cells are considered one of the most critically affected parenchymal cell types. Previous studies have shown that tubulitis, the hallmark histopathological feature of TCMR, predominantly occurs in the proximal tubular compartment, and its severity closely correlates with graft dysfunction and long-term prognosis (5, 19). However, emerging evidence suggests that alterations in proximal tubular cells during TCMR are not primarily driven by direct immune-mediated cytotoxic injury but rather reflect functional remodeling and phenotypic state transitions.

**Figure 2:**
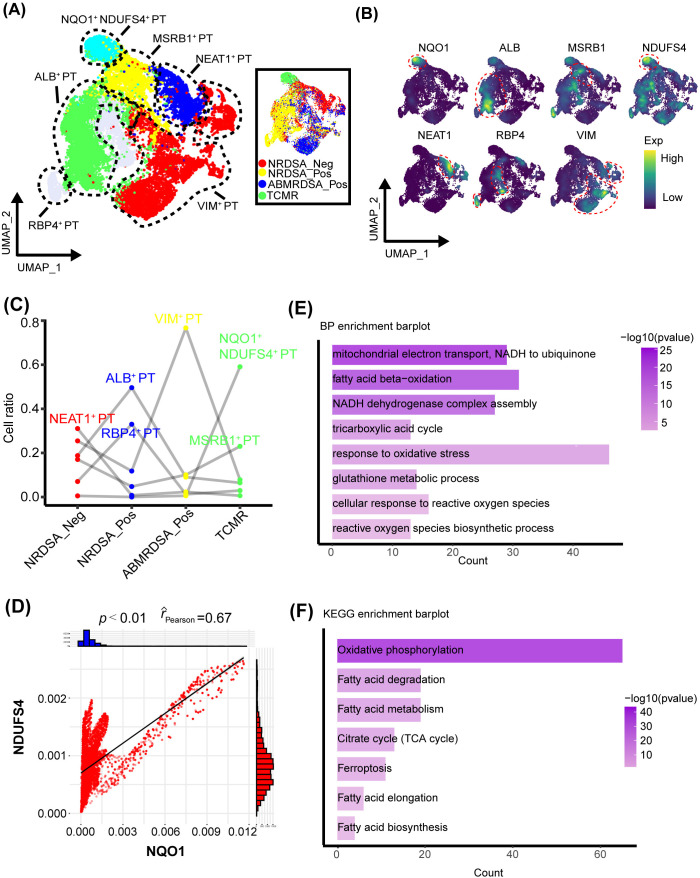
Identification and functional characterization of proximal tubule (PT) cell subsets in TCMR. **(A)** UMAP visualization of proximal tubule (PT) cells extracted from the integrated single-cell dataset. PT cells were further subdivided into transcriptionally distinct subsets based on the expression of characteristic marker genes, including ALB^+^, RBP4^+^, NEAT1^+^, VIM^+^, MSRB1^+^, and NQO1^+^NDUFS4^+^ PT subsets. Colors indicate different clinical groups. **(B)** Feature plots showing the expression patterns of representative marker genes defining each PT cell subset, including NQO1, ALB, MSRB1, NDUFS4, NEAT1, RBP4, and VIM. Color intensity reflects relative gene expression levels. **(C)** Line plot illustrating the relative proportions of PT cell subsets across different clinical groups, including NRDSA-negative, NRDSA-positive, ABMRDSA-positive, and TCMR samples. **(D)** UMAP-based co-expression analysis of NQO1 and NDUFS4 in PT cells. Cells co-expressing both genes are highlighted, illustrating the spatial distribution of the NQO1^+^NDUFS4^+^ PT subset. **(E)** Bar chart displaying GO biological processes significantly enriched in the NQO1^+^NDUFS4^+^ PT subset. **(F)** Bar chart displaying KEGG pathways significantly enriched in the NQO1^+^ NDUFS4^+^ PT subset.

To dissect proximal tubular cell heterogeneity, unsupervised clustering analysis was performed on PT cells extracted from the integrated dataset, identifying six transcriptionally distinct subpopulations, including ALB^+^, RBP4^+^, NEAT1^+^, VIM^+^, MSRB1^+^, and NQO1^+^NDUFS4^+^ PT subsets (**Figure 2A**). Each subpopulation exhibited a unique marker gene expression profile, with NQO1 and NDUFS4 showing marked highexpressed in the NQO1^+^NDUFS4^+^ PT subset (**Figure 2B**). Compared with DSA-negative non-rejection controls, this subset was selectively enriched in TCMR samples and became the dominant PT population (**Figure 2C**). Co-expression analysis revealed highly concordant expression of NQO1 and NDUFS4 within this subset, visualized by UMAP-based highlighting of double-positive cells (**Figure 2D**). Previous studies have reported that NQO1 can function as an RNA-binding protein, directly binding to NDUFS4 transcripts to enhance their stability and promote translation (20), providing a plausible molecular basis for their coordinated regulation.

**Figure 3:**
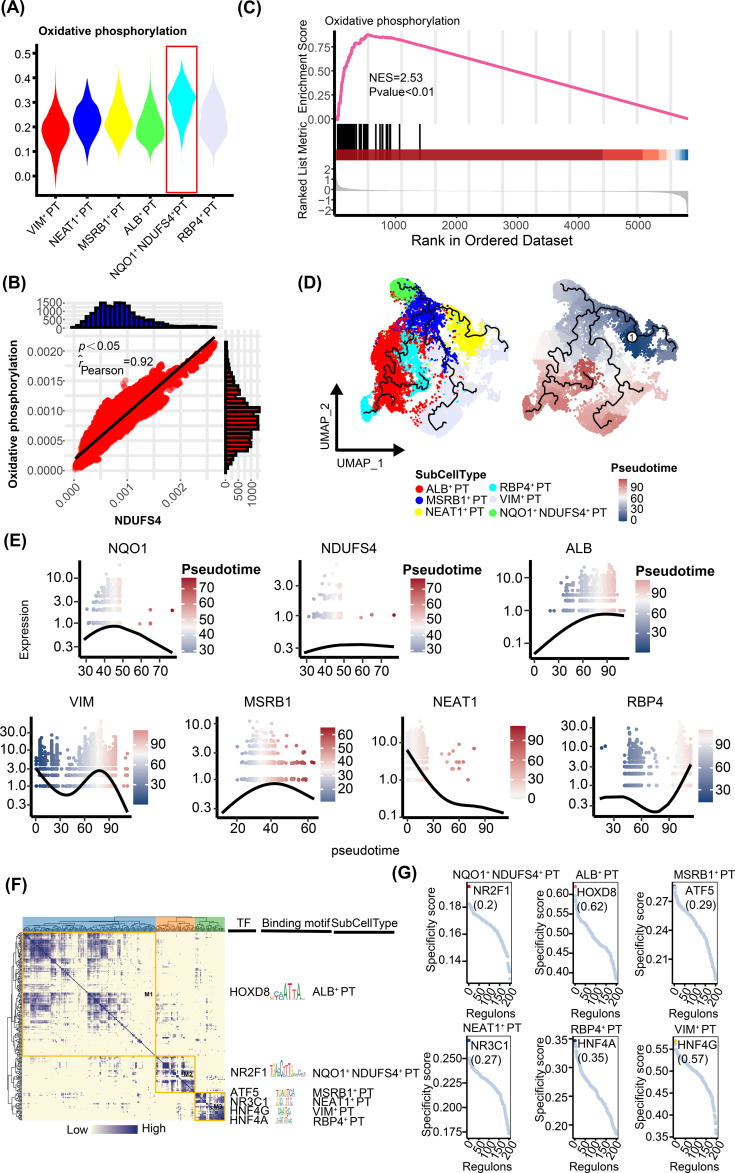
Metabolic activity, pseudotime dynamics, and gene regulatory network analysis of PT cell subsets in TCMR. **(A)** Violin plot showing that the NQO1^+^NDUFS4^+^ PT subset exhibits significantly higher oxidative phosphorylation pathway activity scores compared to other PT subsets. **(B)** Strong positive correlation between NDUFS4 expression and oxidative phosphorylation pathway activity at the single-cell level (Pearson correlation analysis). **(C)** Gene set enrichment analysis (GSEA) shows significant enrichment of the oxidative phosphorylation pathway in the NQO1^+^NDUFS4^+^ PT subset (NES = 2.53, P < 0.01). **(D)** Pseudotime trajectory analysis reveals dynamic transcriptional transitions among PT cell subsets. Cells are colored by subtype (top) or pseudotime value (bottom). **(E)** Expression dynamics of PT marker genes (NQO1, NDUFS4, ALB, VIM, MSRB1, NEAT1, RBP4) along pseudotime, highlighting stage-specific regulation. **(F)** Gene regulatory network analysis reveals differential transcription factor motif activity across PT cell subsets. Heatmap displays distinct regulon modules and their associated transcription factors. **(G)** Specificity scores of key transcription factor regulons across PT subsets, identifying dominant regulators for each subset.

Functional enrichment analysis revealed that the NQO1^+^NDUFS4^+^ PT subset was significantly enriched in oxidative stress–related biological processes (**Figure 2E**) as well as KEGG pathways including oxidative phosphorylation, fatty acid metabolism, and ferroptosis (**Figure 2F**). Oxidative phosphorylation pathway scoring showed that this subset exhibited the highest activity among all PT populations (**Figure 3A**). Correlation analysis indicated that NDUFS4 expression was positively correlated with oxidative phosphorylation scores at the single-cell level (**Figure 3B**). Gene set enrichment analysis further confirmed robust activation of the oxidative phosphorylation pathway in this subset (NES = 2.53, P < 0.01; **Figure 3C**). Oxidative phosphorylation is a central metabolic process driving ATP production while generating mitochondrial reactive oxygen species, and its aberrant activation has been closely linked to oxidative stress, ferroptosis, and epithelial dysfunction (21, 22).

To determine the positioning of this subset within the proximal tubular lineage, pseudotime trajectory analysis was performed to reconstruct differentiation trajectories. NEAT1^+^ PT cells were predominantly located at the beginning of the trajectory, whereas the NQO1^+^NDUFS4^+^ subset was distributed in the intermediate stage (**Figure 3D**), indicating that it does not represent a terminally damaged state but rather a transitional state associated with functional remodeling. Dynamic changes in marker gene expression along pseudotime further supported distinct transcriptional programs among the subpopulations (**Figure 3E**).

Single-cell regulatory network inference analysis further revealed the transcriptional regulatory characteristics of this subset. PT cells were divided into three major regulatory modules based on transcription factor motif activity (**Figure 3F**), among which NR2F1 was identified as a core regulator of the NQO1^+^NDUFS4^+^ subset, exhibiting selectively enhanced transcriptional activity (**Figure 3G**). Given that NR2F1 has been implicated in cell fate maintenance and dedifferentiation (23), its activation further supports the notion that NQO1^+^NDUFS4^+^ PT cells represent a non-terminal, highly plastic reprogrammed state.

Collectively, these data reveal a metabolically reprogrammed NQO1^+^NDUFS4^+^ PT cell subset in the single TCMR sample analyzed. Whether this subset is specifically associated with TCMR requires confirmation in larger studies. Notably, this subset, instead of manifesting overt classical cell death features, exhibits marked enhancement of oxidative phosphorylation, suggesting an adaptive metabolic state induced by inflammatory stress. Previous studies have proposed that proximal tubular alterations in TCMR are characterized not by direct immune cytotoxicity but by functional gene loss, re-expression of embryonic programs, and epithelial dedifferentiation. Tubulitis is thought to result from epithelial barrier disruption that permits passive lymphocyte infiltration rather than direct cytotoxic contact (5). Given the strong dependence of PT cells on fatty acid oxidation, the enrichment of fatty acid metabolism and ferroptosis-related pathways further supports the presence of profound metabolic remodeling and oxidative stress accumulation in this subset. These observations are consistent with the emerging model and suggest metabolic remodeling as a feature of PT cells in TCMR.

### 3.3 The S100A8+ macrophage subset recruits and activates immune cells through chemokine signaling pathways

**Figure 4:**
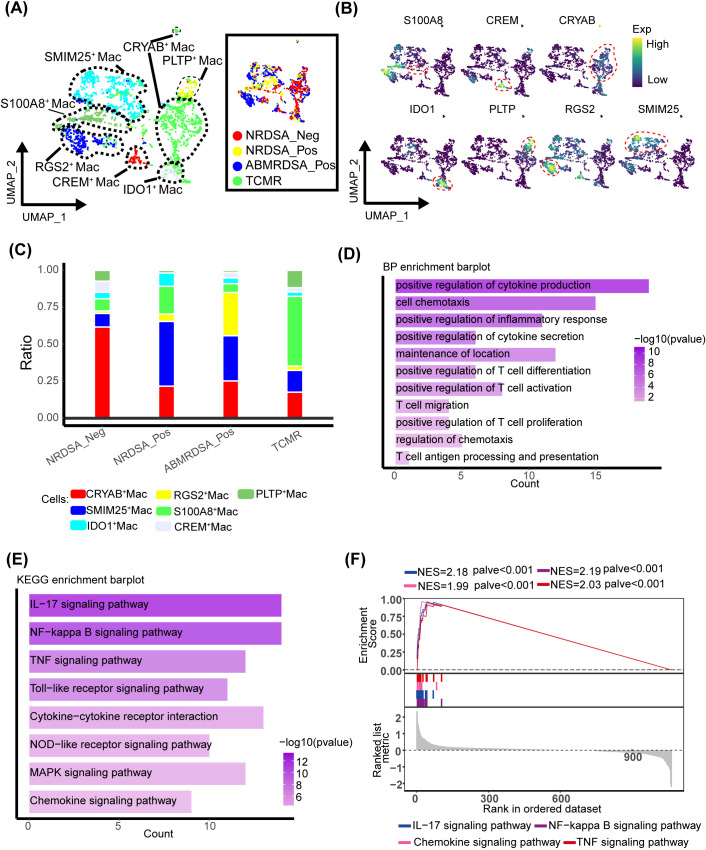
Identification and functional characterization of macrophage (Mac) subsets in TCMR. **(A)** UMAP visualization of macrophage subsets identified in TCMR. Colors indicate different clinical groups. **(B)** Feature plots showing expression patterns of subset-defining marker genes. **(C)** Bar plot illustrating dynamic changes in the relative abundance of macrophage subsets across different clinical groups. **(D)** GO biological process enrichment analysis of macrophage subsets. **(E)** KEGG pathway enrichment analysis of macrophage subsets. **(F)** GSEA enrichment plot for the S100A8^+^ macrophage subset.

Macrophages are no longer considered passive bystanders during T cell–mediated rejection; rather, their infiltration levels closely correlate with rejection severity and graft outcomes, and they play pivotal roles in both acute and chronic rejection (24). To characterize macrophage heterogeneity, unsupervised clustering identified seven transcriptionally distinct macrophage subsets (**Figure 4A**), each with specific marker gene expression profiles (**Figure 4B**). Compared with DSA-negative non-rejection controls, the S100A8^+^ macrophage subset was significantly enriched in TCMR samples and emerged as the dominant macrophage population (**Figure 4C**).

**Figure 5:**
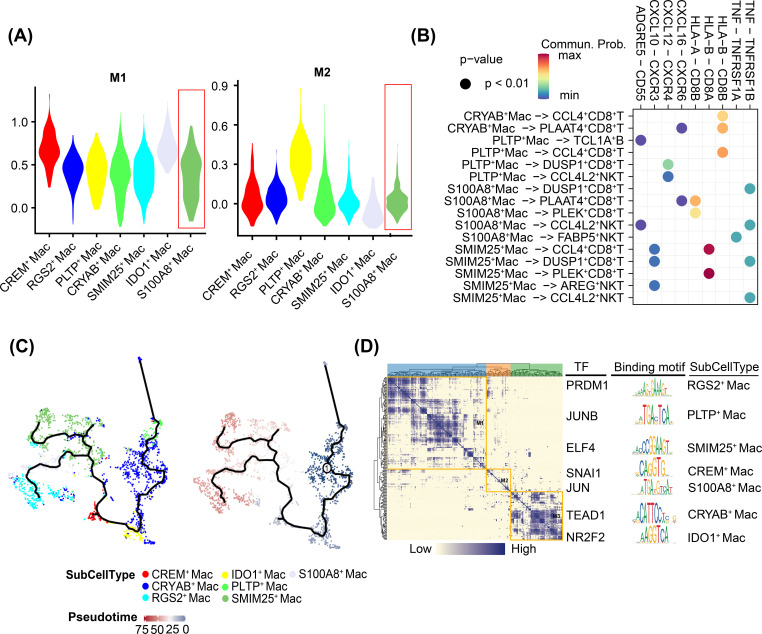
Functional states, cell–cell communication, and regulatory programs of macrophage subsets in TCMR. **(A)** Violin plots showing M1- and M2-like polarization scores across macrophage subsets, indicating functional heterogeneity. **(B)** Bubble plot displaying significant ligand–receptor pairs between different cell-type pairs. Bubble color denotes communication probability, reflecting interaction strength or likelihood. **(C)** Pseudotime trajectory analysis of macrophage subsets revealing dynamic state transitions, visualized by subset annotation and pseudotime progression. **(D)** Heatmap of transcription factor motif activity from gene regulatory network analysis, showing subset-specific regulon modules and key transcription factors.

S100A8 is a low–molecular weight calcium-binding protein predominantly expressed in myeloid cells, often forming heterodimers with S100A9 (25). It amplifies inflammatory responses by activating NF-κB and other signaling pathways, thereby inducing the expression of multiple chemokines and inflammatory cytokines (26, 27). Functional enrichment analysis showed that the S100A8^+^ macrophage subset was significantly enriched in chemokine signaling as well as classical inflammatory pathways. GO biological process analysis revealed enrichment of immune activation-related processes (**Figure 4D**). KEGG pathway analysis confirmed enrichment of NF-κB, IL-17, TNF, Toll-like receptor, and chemokine signaling pathways (**Figure 4E**). Gene set enrichment analysis further confirmed robust activation of these inflammatory signaling pathways in the S100A8^+^ macrophage subset (**Figure 4F**). Consistently, this subset exhibited a typical M1-like polarization phenotype, with significantly higher inflammatory response scores compared with other macrophage subsets (**Figure 5A**), in line with previous reports highlighting the dominant role of inflammatory macrophages in TCMR (6, 28).

Cell–cell communication analysis revealed that S100A8^+^ macrophages formed extensive interaction networks with NKT cells and CD8^+^ T cells. These networks involved multiple signaling axes, including chemokine pathways (CXCL10–CXCR3, CXCL12–CXCR4, CXCL16–CXCR6), antigen presentation (HLA/B–CD8A/CD8B), and inflammatory signaling (TNF–TNFRSF1A/B) (**Figure 5B**). Previous studies have demonstrated that macrophage-derived chemokines are essential for T cell recruitment, positioning, and effector function enhancement (29). In addition, macrophages can act as non-classical antigen-presenting cells, potentially enhancing CD8^+^ T cell activation through MHC class I pathways and amplifying rejection responses (30). The observed ligand–receptor pairings through HLA/B–CD8A/CD8B and TNF–TNFRSF1A/B are consistent with this possibility. This raises the hypothesis that S100A8^+^ macrophages may not only recruit T cells but could also provide activation signals. However, direct functional evidence is required to confirm such a role.

Pseudotime trajectory analysis revealed that CRYAB^+^ macrophages were located at the beginning of the differentiation trajectory, whereas S100A8^+^ macrophages primarily occupied an intermediate stage (**Figure 5C**), suggesting an inflammation-driven activation transition. Single-cell regulatory network inference identified JUN as the core transcriptional regulator of this subset (**Figure 5D**). JUN, a key component of the AP-1 complex, has been shown to play a central role in inflammatory macrophage activation and chemokine regulation (31, 32).

In summary, our bioinformatics analyses identify a highly pro-inflammatory S100A8^+^ macrophage subset in the TCMR microenvironment. This subset exhibits M1-like polarization, shows strong enrichment in chemokine signaling, and is predicted to recruit T cells and NKT cells via chemokine signaling and potentially promote CD8^+^ T cell activation through MHC class I–mediated antigen presentation. These findings, derived from a single TCMR case, suggest a potential role for this subset in contributing to the amplification and persistence of local immune responses. Direct functional evidence and replication in independent TCMR samples are required.

### 3.4 DUSP1^+^ effector CD8^+^ T cells may contribute to rejection through T cell receptor signaling

CD8^+^ cytotoxic T lymphocytes are recognized as key effector cells potentially contributing to graft injury during TCMR. Persistent alloantigen stimulation further drives CD8^+^ T cells toward a highly activated effector state, enabling them to maintain tissue-damaging capacity within chronic inflammatory microenvironments (33). However, the functional heterogeneity of CD8^+^ T cells in TCMR has not been comprehensively characterized.

**Figure 6:**
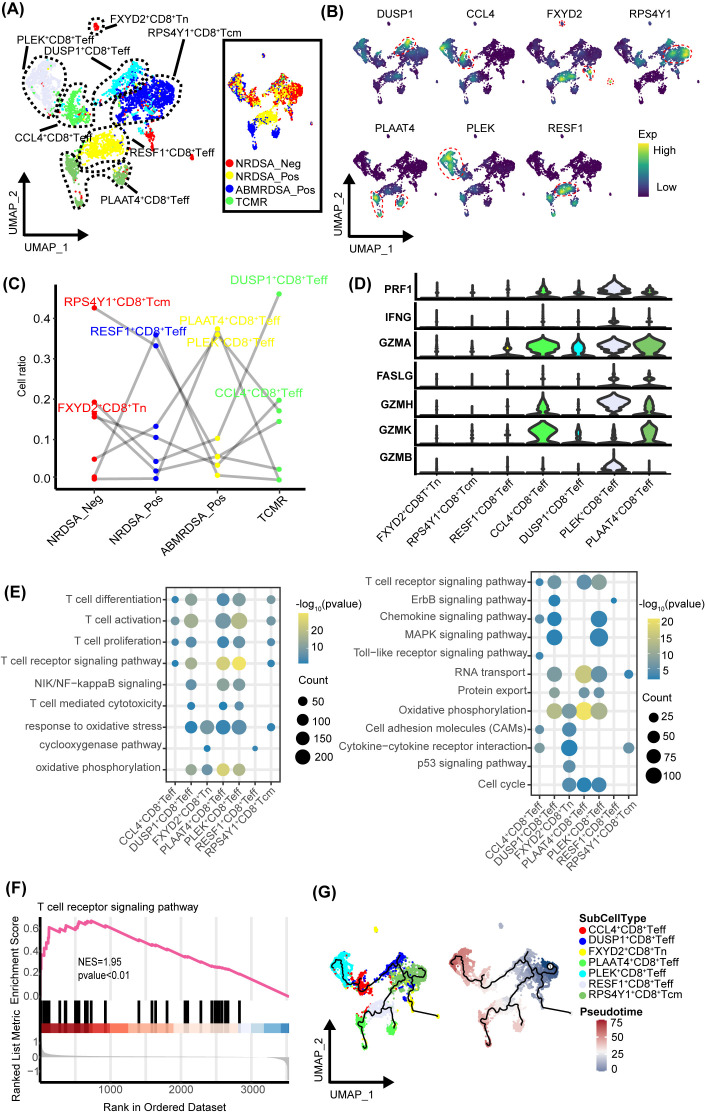
Identification, functional characterization, and trajectory analysis of CD8^+^ T cell subsets in TCMR. **(A)** UMAP visualization of CD8^+^ T cell subsets identified in TCMR, including CCL4^+^, DUSP1^+^, PLAAT4^+^, PLEK^+^, and RESF1^+^ effector T cells, FXYD2^+^ CD8^+^ naïve T cells (Tn), and RPS4Y1^+^ central memory T cells (Tcm). **(B)** Feature plots showing expression patterns of subset-defining marker genes (DUSP1, CCL4, FXYD2, RPS4Y1, PLAAT4, PLEK, RESF1). **(C)** Line plot illustrating dynamic changes in the proportions of CD8^+^ T cell subsets across different clinical groups. **(D)** Violin plots showing expression differences of cytotoxicity-related genes across distinct CD8^+^ T cell subsets. **(E)** Functional enrichment analysis of CD8^+^ T cell subsets, revealing GO biological processes related to T cell-mediated cytotoxicity, immune effector function, T cell activation, and IFN-γ production, as well as KEGG pathways including T cell receptor, chemokine, MAPK, NF-κB signaling, and oxidative phosphorylation. **(F)** Gene set enrichment analysis (GSEA) indicates significant activation of the T cell receptor signaling pathway in the DUSP1^+^ effector CD8^+^ T cell subset (NES = 1.95, P < 0.01). **(G)** Pseudotime trajectory analysis of CD8^+^ T cells revealing dynamic differentiation trajectories, visualized by subset annotation (left) and pseudotime progression (right).

Unbiased clustering of intragraft CD8^+^ T cells identified seven transcriptionally distinct subsets (**Figure 6A**). Based on canonical marker expression, clusters with high expression of DUSP1, PLAAT4, PLEK, CCL4, and RESF1 were defined as effector CD8 ^+^ T cells. The RPS4Y1 ^+^ cluster was classified as central memory CD8 ^+^ T cells (Tcm). The FXYD2 ^+^ cluster was defined as naïve-like CD8 ^+^ T cells (Tn) (**Figure 6B**; **Supplementary Figure 1A**). Notably, PLAAT4^+^ and PLEK^+^ effector CD8^+^ T cells were more abundant in ABMR samples, whereas the DUSP1^+^ effector CD8^+^ T cell subset was selectively enriched in TCMR (**Figure 6C**).

Cytotoxic effector molecules exhibited subset-specific expression patterns: GZMB and IFNG were mainly expressed in PLEK^+^ effector cells, whereas PRF1, GZMA, and GZMK were expressed across PLAAT4^+^, PLEK^+^, and DUSP1^+^ effector subsets (**Figure 6D**). AUCell scoring further revealed that PLAAT4^+^ and PLEK^+^ effector CD8^+^ T cells exhibited higher cytotoxicity scores, whereas DUSP1^+^ effector and FXYD2^+^ naïve-like CD8^+^ T cells displayed higher activation potential (**Supplementary Figures 1B, C**). Functional enrichment analysis showed that CD8^+^ T cell subsets were enriched in GO biological processes related to T cell-mediated cytotoxicity, immune effector function, T cell activation, and IFN-γ production. In addition, they were enriched in multiple KEGG pathways, including T cell receptor, chemokine, MAPK, NF-κB signaling, and oxidative phosphorylation (**Figure 6E**). Specifically, the DUSP1^+^ effector CD8^+^ T cells were significantly enriched for components of the TCR signaling pathway. Gene set enrichment analysis further confirmed strong activation of the TCR signaling pathway in this subset (NES = 1.95, p < 0.01; **Figure 6F**). These findings are consistent with previous evidence that T cells recognize donor HLA molecules through TCR signaling (34), thereby initiating both direct and indirect pathways of allorecognition, while sustained TCR engagement drives effector differentiation of alloreactive CD8^+^ T cells and promotes graft injury. Therefore, we speculate that the DUSP1^+^ CD8^+^ T cell subset may serve as a key CD8^+^ T cell subset driving TCMR progression.

**Figure 7:**
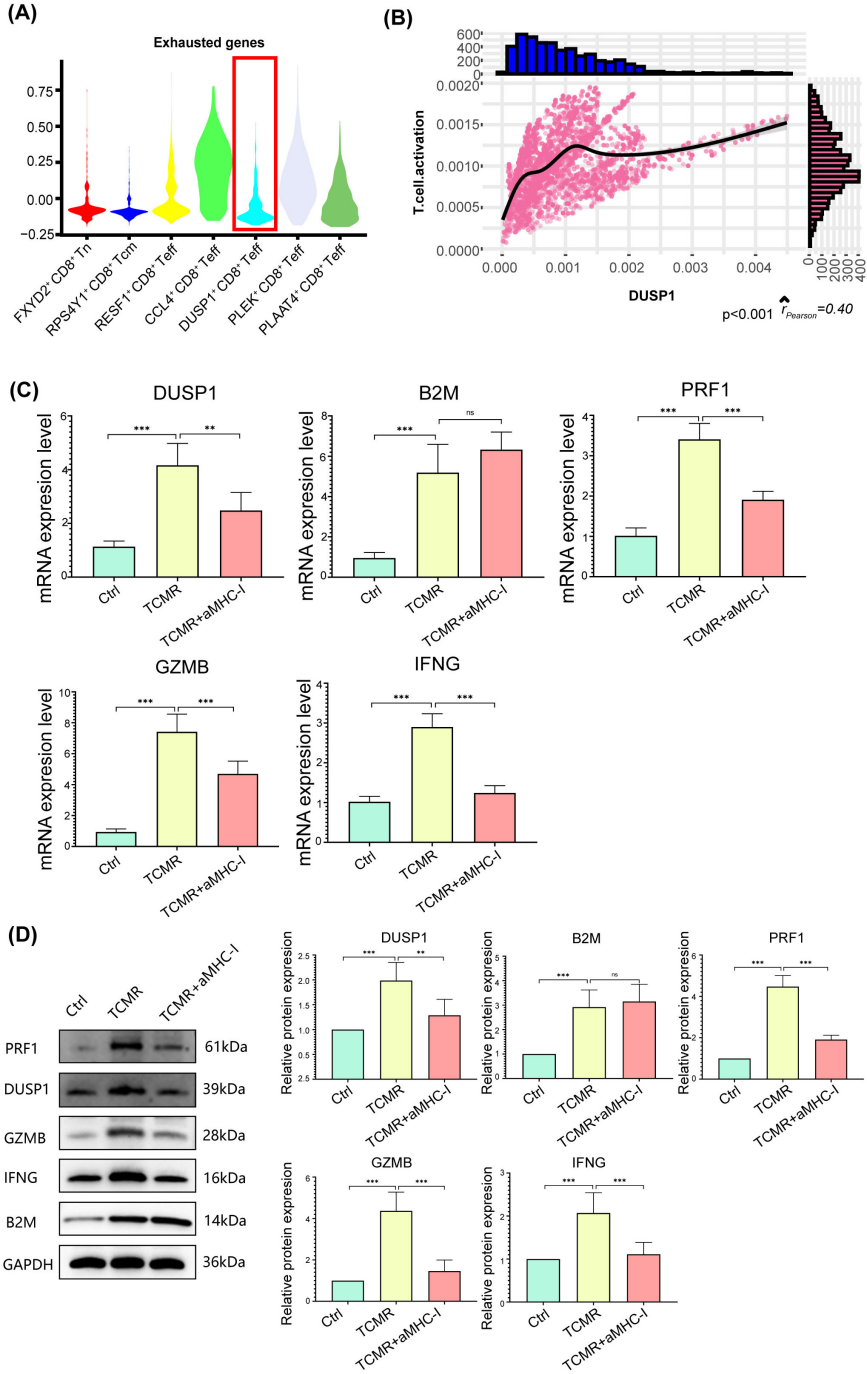
Expression and validation of key molecules in TCMR. **(A)** Expression of exhaustion-associated genes across CD8^+^ T cell subsets. **(B)** Correlation analysis showing a significant positive correlation between DUSP1 expression and T cell activation scores. **(C)** Transcriptional levels of key molecules (Dusp1, B2m, Gzmb, Prf1, Ifng) in the TCMR, Ctrl, and TCMR^+^aMHC-I groups. **(D)** Western Blot validation of the protein expression levels of the above key molecules (DUSP1, B2M, GZMB, PRF1, IFNG) across the three animal groups. ** indicates p < 0.01, *** indicates p < 0.001, and ns indicates not significant.

Pseudotime analysis positioned DUSP1^+^ effector CD8^+^ T cells in an intermediate differentiation state with activated transcriptional features (**Figure 6G**). Exhaustion-associated genes were expressed at relatively low levels across all CD8^+^ T cell subsets (**Figure 7A**), suggesting retained functional activity rather than terminal exhaustion. DUSP1 is a key regulator of TCR signaling. On one hand, DUSP1 functions as a negative feedback regulator that dephosphorylates MAPK components (JNK/ERK) to prevent pathological T cell overactivation (35, 36); on the other hand, its expression is dynamically regulated during T cell activation and differentiation (35). In our data, DUSP1 expression positively correlated with T cell activation scores (**Figure 7B**), suggesting that in the context of TCMR, DUSP1 may serve as a surrogate indicator of CD8^+^ T cell activation status.

To experimentally validate the involvement of DUSP1^+^ effector CD8^+^ T cells in TCMR, we established a murine TCMR model, including syngeneic controls, TCMR, and CD8^+^ T cell activation inhibition groups. Compared with syngeneic controls, TCMR grafts exhibited significant upregulation of Dusp1, B2m, and effector molecules (Gzmb, Prf1, Ifng) at both mRNA and protein levels (**Figures 7C, D**). Importantly, blockade of B2M/MHC class I signaling significantly reduced the expression of these effector genes (**Figures 7C, D**).

Together, these data suggest that the DUSP1-associated activation pathway, particularly MHC class I signaling, is upregulated in TCMR and may represent a candidate for further investigation. Targeting this pathway could be explored as a potential therapeutic strategy for TCMR.

### 3.5 The CCL4L2+ NKT cell subset exacerbates T cell–mediated rejection through chemokine recruitment and cytotoxic effects

**Figure 8:**
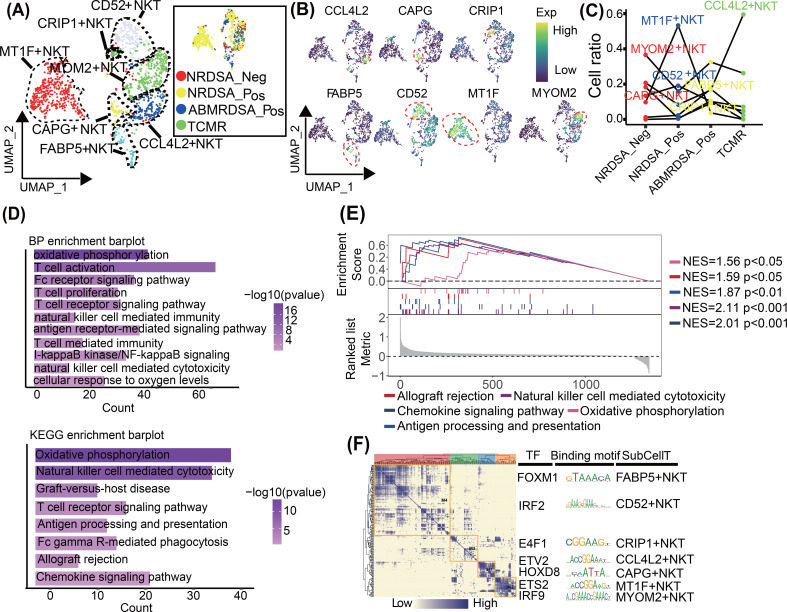
Identification and analysis of natural killer T (NKT) cell subsets in TCMR. **(A)** Single-cell atlas of NKT cell subsets (UMAP visualization). **(B)** Density plot displaying specific marker genes of NKT cell subsets. **(C)** Dot plot showing the NKT cell subset compositions of different groups. **(D)** Bar chart displaying KEGG pathways and GO biological processes significantly enriched in NKT cell subsets. **(E)** GSEA plots showing signaling pathways significantly activated in the CCL4L2^+^ NKT cell subset. **(F)** Heatmap of transcription factor motif activity (SCENIC) showing gene regulatory networks of NKT cell subsets.

Natural killer T (NKT) cells serve as a critical bridge between innate and adaptive immunity by secreting cytokines and chemokines that regulate immune cell migration and activation (37). Unbiased clustering of intragraft NKT cells identified seven transcriptionally distinct subsets (**Figure 8A**), each exhibiting unique gene expression profiles (**Figure 8B**). Compared with DSA-negative non-rejection controls, the CCL4L2^+^ NKT cell subset was significantly enriched in TCMR samples and emerged as the dominant population (**Figure 8C**).

Functional enrichment analysis revealed that this subset was significantly enriched in pathways related to chemokine signaling, natural killer cell–mediated cytotoxicity, and graft-versus-host disease (**Figure 8D**). Gene set enrichment analysis (GSEA) further confirmed robust activation of these pathways in the CCL4L2^+^ NKT cell subset (**Figure 8E**). Single-cell regulatory network analysis identified distinct transcriptional regulatory modules across NKT cell subsets, as illustrated by the heatmap of transcription factor motif activity (**Figure 8F**).

In summary, analysis of the single TCMR sample revealed a CCL4L2^+^ NKT cell subset enriched in that sample, which may possess chemokine-secreting and cytotoxic effector functions. These hypothesis-generating observations suggest that this subset could contribute to local immune amplification, but they must be tested in additional TCMR cases.

## 4 Discussion

T cell–mediated rejection (TCMR) is generally regarded as an immune-driven process that initiates with alloantigen recognition and propagates through T cell activation and cytotoxic injury (38, 39). Although this classical model has been well validated, recent advances in single-cell and spatial transcriptomics have suggested that parenchymal cells are not merely passive targets but actively participate in shaping the rejection microenvironment (40). In this context, the present study, based on a single human TCMR case, integrates immune activation and tissue-intrinsic adaptive processes at single-cell resolution, providing a hypothesis-generating framework for understanding the potential pathogenesis of TCMR. However, as this study is derived from a single TCMR sample, all interpretations regarding TCMR-specific molecular features or cellular subsets are preliminary and should not be generalized without validation in larger independent cohorts.

Traditional views have attributed tubular injury in TCMR mainly to direct cytotoxicity of immune cells (41). However, studies by Philip F. Halloran and colleagues demonstrated that T cell-derived granzymes and perforin are not the key effector molecules in TCMR (42); D. Kayser et al. further confirmed that T cells do not directly cause TCMR injury through Fas-FasL-mediated cytotoxicity (43). These lines of evidence imply that the occurrence of epithelial injury in TCMR may be regulated by indirect mechanisms, i.e., epithelial cells undergo “dedifferentiation” in response to inflammatory stimuli (5, 44). In the single TCMR sample analyzed here, the NQO1^+^NDUFS4^+^ PT subset enriched in TCMR showed a metabolic signature suggesting enhanced energy metabolism and increased susceptibility to oxidative damage, consistent with PT cell adaptive reprogramming (40). The coordinated upregulation of NQO1 and NDUFS4 further suggests that they might constitute a regulatory axis linking redox homeostasis and mitochondrial function, thereby supporting a hypothetical model in which metabolic reprogramming is not merely a concomitant phenomenon of injury but could influence the vulnerability of epithelial cells during rejection. Under inflammatory stress, persistent activation of OXPHOS can lead to excessive mitochondrial ROS (mtROS) production. Indeed, defective mitochondria are the main source of ROS in most cells, with complexes I and III identified as primary sites of ROS production during OXPHOS. Accumulating evidence indicates that mtROS overproduction can trigger ferroptosis, an iron-dependent cell death characterized by lipid peroxidation and glutathione depletion — consistent with the enrichment of ferroptosis-related pathways observed in **Figure 2F**. This mtROS-driven ferroptosis has been implicated in renal tubular injury and ischemia-reperfusion injury following kidney transplantation (21, 45).

Regarding immune cell composition, macrophages have long been recognized as important mediators of inflammatory responses and antigen presentation in transplant rejection, and their infiltration level correlates with rejection severity (44). During transplant rejection, macrophages present antigens to T cells through MHC-II and co-stimulatory molecules CD80/CD86, and simultaneously secrete IL-12 and IL-23 to promote T cell maturation and function (46). Consistent with this, our observation in the single TCMR case suggests that S100A8^+^ macrophages may serve as potential coordinators of the immune amplification network. S100A8 is a pro-inflammatory molecule that activates NF-κB signaling (47), and the S100A8^+^ macrophage subset in TCMR exhibited an M1-like phenotype. Moreover, S100A8^+^ macrophages were predicted to interact with CD8^+^ T cells and NKT cells through chemokine axes such as CXCL10–CXCR3 and CXCL12–CXCR4, as well as the inflammatory signaling pathway TNF–TNFRSF1A/B, which could amplify immune responses. In parallel, recent spatial transcriptomic studies have highlighted FCGR3A^+^ monocytes/macrophages as another innate immune subset potentially involved in acute kidney transplant rejection (24). While the present study focused on S100A8^+^ macrophages, the potential contribution of FCGR3A^+^ cells to TCMR merits further investigation.

Given the dominant role of T cells in TCMR, a 2018 review highlighted that the strength and duration of TCR signals are key determinants of T cell effector function (48). In the present single TCMR case, the DUSP1^+^ effector CD8^+^ T cell subset enriched in TCMR exhibited a highly activated TCR-signaling phenotype. DUSP1 plays a dual role in T cell function. On one hand, as a MAPK phosphatase, DUSP1 limits excessive T cell activation by dephosphorylating JNK/ERK, exerting a classical negative feedback regulatory function (35, 36); on the other hand, DUSP1 is equally indispensable for full T cell activation, as its presence permits NFATc1 nuclear translocation and initiates TCR signaling. Based on this, it is possible that the pathogenicity of DUSP1^+^ effector CD8^+^ T cells in TCMR may not arise from the “negative regulatory” function of DUSP1, but rather from its role as a “permissive factor” that enables full T cell activation. Under persistent alloantigen stimulation, DUSP1 expression could facilitates sustained TCR signal transduction, thereby supporting CD8^+^ T cells to maintain a highly activated effector state and subsequently exert immune rejection. Finally, we also identified an NKT cell subset possessing both chemotactic and cytotoxic functions. As innate-like lymphocytes bridging innate and adaptive immunity, NKT cells might amplify local immune responses by promoting immune cell recruitment and directly exerting effector functions.

Integrating the above findings, we propose a hypothetical multi-cellular synergistic model of TCMR in which macrophages recruit and organize effector lymphocytes through chemokine signals, NKT cells further amplify local immune responses, CD8^+^ T cells mediate tissue injury through antigen-dependent cytotoxic mechanisms, and parenchymal cells modulate their susceptibility to injury via metabolic adaptation. This framework moves beyond the traditional immune-centric model by incorporating tissue-intrinsic responses as key determinants, but it requires validation in larger TCMR cohorts and functional experiments.

The present study has several limitations. Importantly, the TCMR-related findings are derived from a single human TCMR sample; therefore, all interpretations regarding TCMR-specific molecular features or cellular subsets are hypothesis-generating in nature and should not be considered generalizable. The integrated analysis and murine model provide supportive contextual data, but independent replication in larger human TCMR cohorts is essential. In addition, differences between the mouse model and the human immune system must be interpreted with caution. Future studies integrating larger cohorts, spatial transcriptomics, and functional experiments are needed to further validate and refine this model.

## 5 Conclusion

In conclusion, this hypothesis-generating study, based on a single TCMR case, provides a preliminary single-cell atlas of the TCMR immune microenvironment. Our findings highlight the DUSP1-associated activation pathway and its associated B2M/MHC class I axis as a candidate pathway for therapeutic exploration. Additionally, the S100A8^+^ macrophage subset may serve as a potential coordinator of immune amplification, interacting with CD8^+^ T cells through chemokine and inflammatory signals. These insights suggest a framework linking innate-adaptive crosstalk to allograft injury that warrants further investigation in larger cohorts and functional studies.

## Data Availability

The datasets analyzed for this study can be found in the NCBI Repository; Accession number GSE145927 and in the functional genomics data collection (ArrayExpress), Accession number E-MTAB-12051.
