# Supplementary material for: Innate-immune crosstalk orchestrates T cell-mediated rejection in kidney transplants
Source: Front Immunol. 2026 Jul 7;17:1827224. doi: 10.3389/fimmu.2026.1827224 (PMC13384830; doi:10.3389/fimmu.2026.1827224)
Supplement: Supplementary file 3 [file Image1.pdf]

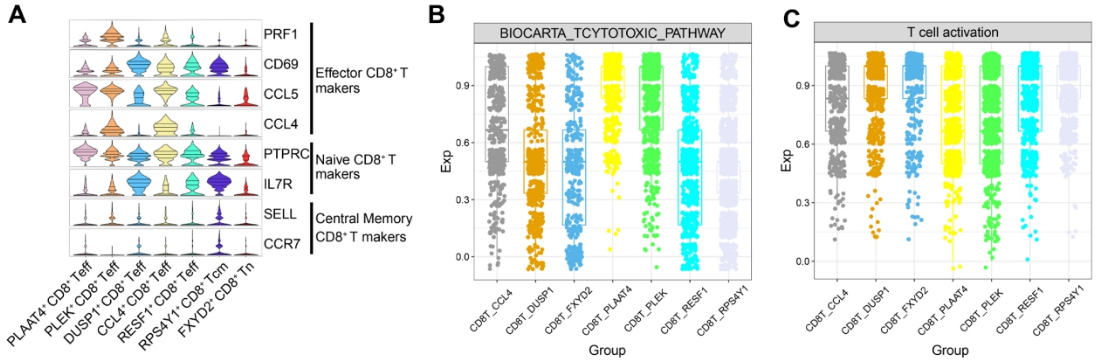

Figure S1. Canonical markers and functional scoring of CD8⁺ T cell subsets.

A.Expression patterns of canonical marker genes across CD8⁺ T cell subsets, including effector CD8⁺ T (Teff), central memory CD8⁺ T (Tcm), and naïve CD8⁺ T (Tn) cells.

B. Boxplot showing cytotoxic pathway activity scores calculated by AUCell based on the BIOCARTA_TCYTOTOXIC_PATHWAY gene set across different CD8⁺ T cell subsets.

C.Boxplot showing T cell activation signature scores calculated by AUCell across different CD8⁺ T cell subsets. Each dot represents a single cell, and the box indicates the interquartile range with median values.
